# Association of Adenovirus 36 Infection with Obesity and Metabolic Markers in Humans: A Meta-Analysis of Observational Studies

**DOI:** 10.1371/journal.pone.0042031

**Published:** 2012-07-25

**Authors:** Tomohide Yamada, Kazuo Hara, Takashi Kadowaki

**Affiliations:** Department of Diabetes and Metabolic Diseases, Graduate School of Medicine, University of Tokyo, Japan; University of Verona, Ospedale Civile Maggiore, Italy

## Abstract

**Background:**

Several studies have shown that Adenovirus 36 (Ad36) influences the risk of obesity in humans. Clarifying the relationship between Ad36 infection and obesity could lead to more effective approaches for the management of obesity. The objective of this study was to conduct a meta-analysis to confirm the influence of Ad36 infection on obesity and metabolic markers.

**Methodology/Principal Findings:**

We searched MEDLINE and the Cochrane Library for pertinent articles (including their references) published between 1951 and April 22, 2012. Only English language reports of original observational studies were included in this meta-analysis. Data extraction was performed independently by two reviewers. Weighted mean differences (WMDs) and pooled odds ratios (ORs) with 95% confidence intervals (95% CIs) were calculated using the random effects model. Of 237 potentially relevant studies, 10 cross-sectional studies (n = 2,870) conformed to the selection criteria. Pooled analysis showed that the WMD for BMI of Ad36 infection compared with non-infection was 3.19 (95% CI 1.44–4.93; P<0.001). Sensitivity analysis restricted to studies of adults yielded a similar result of 3.18 (95% CI 0.78–5.57; P = 0.009). The increased risk of obesity associated with Ad36 infection was also significant (OR: 1.9; 95% CI: 1.01–3.56; P = 0.047). No significant differences were found in relation to total cholesterol (P = 0.83), triglycerides (P = 0.64), HDL (P = 0.69), blood glucose (P = 0.08), waist circumstance (P = 0.09), and systolic blood pressure (P = 0.25).

**Conclusion/Significance:**

Ad36 infection was associated with the risk of obesity and weight gain, but was not associated with abnormal metabolic markers including waist circumstance. It suggests that Ad36 infection is more associated with accumulation of subcutaneous fat than that of visceral fat. The relationship between Ad36 and obesity should be assessed by further studies, including well-designed prospective studies, to gain a better understanding of whether Ad36 plays a role in the etiology of human obesity.

## Introduction

Obesity has rapidly become a worldwide epidemic, with one billion people being either overweight or obese around the world [1]. Obesity is a major risk factor for many serious disorders, such as diabetes, cardiovascular disease, and cancer [2]. A number of large-scale epidemiologic studies have evaluated the relationship between obesity and mortality [3], [4], revealing that a high body mass index (BMI) is associated with an increased rate of death from all causes and from cardiovascular disease. Most management strategies for obesity target the behavioral component of this disorder (e.g., by lifestyle modification), but are minimally effective. Thus, new therapeutic strategies are required.

Several recent studies have shown that Adenovirus 36 (Ad36) is associated with the risk of obesity and weight gain in humans [5]–[14]. These studies have generally found a positive association of Ad36 infection with obesity, although its magnitude has varied. Clarifying the relationship between Ad36 infection and obesity could lead to more effective approaches for the management of this condition. Accordingly, we performed a meta-analysis to confirm the influence of Ad36 infection on obesity and metabolic markers.

## Methods

### Searches

The Medline and Cochrane Library electronic databases were searched from 1951 until April 22, 2012 using the medical subject headings (MeSH) “Adenovirus 36” and “Obesity” to identify observational studies that tested the association between Ad36 and obesity, BMI, and other metabolic markers in humans. Reference lists of the pertinent articles were also reviewed.

### Selection

We performed initial screening of study titles or abstracts, while the second screening was based on full-text review. Cohort studies, case-control studies, and cross-sectional studies evaluating the relation of obesity to Ad36 infection were considered eligible for inclusion if the following criteria were fulfilled: 1) the full text of report was published in English; 2) event numbers in each exposure category were reported; 3) the presence of Ad36 infection was reported; and 4) obesity events and/or BMI and other metabolic markers were reported.

### Assessment of Validity

To ascertain the validity of the eligible studies, the quality of each report was appraised with reference to the STROBE statement [15].

### Data Extraction

Two independent investigators (T.Y. and K.H.) reviewed each study to determine its eligibility and then extracted and tabulated all of the relevant data. Disagreement was resolved by consensus between the two reviewers.

The following information was obtained from each study: first author, year of publication, type of subjects, country, parameters (obesity, BMI, triglycerides (TG), total cholesterol (TC), low-density lipoprotein (LDL), high-density lipoprotein (HDL), blood glucose (BG), systolic blood pressure (SBP), waist circumstance (WC)), assessment of obesity, number of subjects, mean age, proportion of females, mean BMI, participants, and prevalence of Ad36 infection. Numerical data from the reports were used, and other data required for analysis (e.g., the mean±SD for the Ad36-positive and Ad36-negative groups) were calculated from the numerical data. The authors of the studies were contacted if necessary to obtain further details.

### Quantitative Synthesis of Data

The weighted mean difference (WMD) and pooled odds ratio (OR) were calculated to evaluate the association of Ad36 with obesity, BMI, and other metabolic markers across the studies by DerSimonian-Laird random effects meta-analysis. We also performed sensitivity analyses restricted to studies of adults, studies of children, and studies performed in the USA, in the EU, or in Asia. Univariate and multivariate meta-regression analyses were performed to explore sources of heterogeneity. Variables such as the type of subject (adults or children), sex (proportion of females), and geographic region (USA, EU, or Asia) were examined for a significant influence on the risk of obesity.

The Cochrane χ^2^ test and the I2 test were used to evaluate heterogeneity among studies, and a threshold value of p = 0.10 was considered to be significant [16]. The possibility of publication bias was evaluated by creating a funnel plot of the effect size for each study versus the SE. Funnel plot asymmetry was assessed by the Begg and Egger tests. All statistical analyses were performed with Stata 11.0 software (StataCorp, College Station, TX). Results are expressed as the mean with 95% CI, unless otherwise indicated. Except for tests of heterogeneity, a P value of less than 0.05 was considered significant. All procedures were performed in accordance with the guideline for the meta-analysis of observational studies in epidemiology [17] and the PRISMA statement [18].

## Results

### Search Results

**Figure 1:**
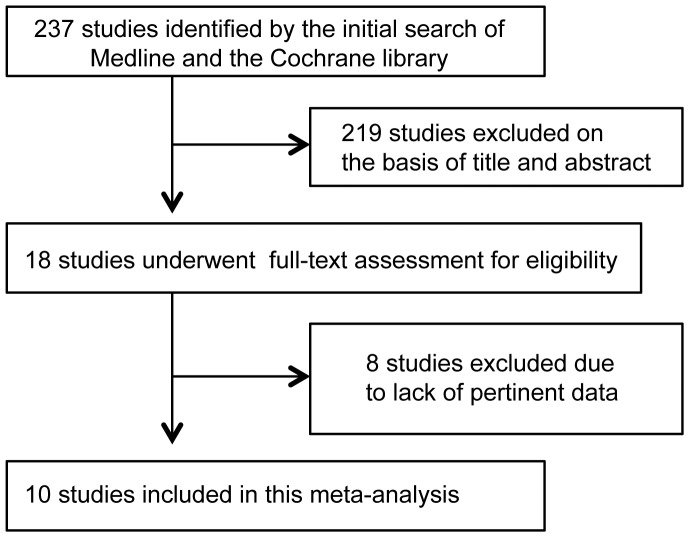
Flow diagram of study selection.

**Figure 1** shows a flow chart of study selection. We identified a total of 237 reports by the database searches. Of these, 219 reports were excluded after review of the title and abstract, leaving 18 for further evaluation. A further eight studies were excluded after full-text evaluation, chiefly because these studies did not contain pertinent data. A total of 10 studies [5]–[14] eventually fulfilled the inclusion criteria and were used in this meta-analysis.

### Study Characteristics

All 10 studies selected were cross-sectional studies and **Table 1** shows their characteristics. There was moderate heterogeneity of the country of origin, mean age, proportion of females, and mean BMI. The studies were published between 2005 and 2012. Seven studies [5], [6], [8], [11]–[14] investigated adults and three [7], [9], [10] were performed on children. Four studies [6], [11], [12], [14] were conducted in the EU, three [5], [8], [9] in the USA, and three [7], [10], [13] in Asia. The size of the study populations ranged from 56 to 540 subjects (mean: 261 subjects). The mean age, proportion of females, and mean BMI were generally in the range between 40–45 years for studies of adults (11–14 years for studies of children), 40–70%, and 24–28 kg/m^2^.

**Table 1:** Summary of studies evaluating the association between Adenovirus 36 (Ad36) infection and obesity/metabolic markers in humans.

First author, year	Type ofsubject	Country	Parameters	Assessmentof obesity	Number of subjects	Mean age (yrs)	Female (%)	Mean BMI(kg/m^2^)	Participants	Prevalence of Ad36 infection
Atkinson, 2005 (5)	Adults	USA	Obesity, BMI, TG, TC	BMI≧30	502	40.9	78	38.0	360 obese and 142non-obese adults	Obese 30% Non-obese 11%
Atkinson, 2005 (5)	Adults	USA	BMI, TG, TC	NA	56	NA	79	25.3	28 sets of twins	Overall 22%
Trovato, 2009 (6)	Adults	Italy	Obesity, BMI, TG, TC, LDL,HDL, SBP	BMI>30	203	45.9	64	27.7	68 obese and 135non-obese adults	Obese 65% Non-obese 33%
Atkinson, 2010 (7)	Children	South Korea	TC, WC, SBP, BG	NA	84	14.8	16	NA	83 obese or overweightchildren and one non-obese child	Overall 30%
Broderick, 2010 (8)	Adults	USA	Obesity	BMI>29	293	NA	30	NA	146 obese and 147non-obese adults	Obese 34% Non-obese 39%
Gabbert, 2010 (9)	Children	USA	Obesity, BMI, WC	BMI>95th percentile	124	13.4	44	27.9	67 obese and 57 non-obese children	Obese 22% Non-obese 7%
Na, 2010 (10)	Children	South Korea	Obesity, BMI, TG, TC, WC,LDL, HDL, SBP, BG	BMI≧30	318	11.7	42	24.1	259 obese and 59 non-obese children	Obese 29% Non-obese 14%
Trovato, 2010 (11)	Adults	Italy	BMI, TG, TC, LDL, HDL, BG	NA	179	45.7	65	27.4	65 NAFLD and 114 non-NAFLD adults	NAFLD 32% Non-NAFLD 46%
Goossens, 2011 (12)	Adults	Netherlands and Belgium	Obesity, BMI	NA	509	NA	NA	26.8	136 obese, 281 non-obese,and 92 BMI-unknownadults	Obese 6% Non-obese 4%
Na, 2012 (13)	Adults	South Korea	Obesity, BMI, TG, TC, WC,HDL, SBP, BG	BMI≧25	540	44.3	50	24.0	180 obese and 360non-obese adults	Obese 30% Non-obese 36%
Trovato, 2012 (14)	Adults	Italy	BMI, TG, TC, LDL, HDL, BG	NA	62	50	56	30.3	62 NAFLD adults	Overall 40%

BMI, body mass index (calculated as weight in kilograms divided by height in meters squared); TG, triglycerides; TC, total cholesterol; LDL, low density lipoprotein; HDL, high density lipoprotein; BG, blood glucose; NAFLD, Non-alcoholic fatty liver disease; SBP, systolic blood pressure; WC, waist circumstance; NA, not available;

Among the 10 studies, eight reported [5], [6], [9]–[14] the BMI, seven [5]–[7], [10], [11], [13], [14] reported total cholesterol (TC) levels, seven [5], [6], [8]–[10], [12], [13] reported about obesity, six [5], [6], [10], [11], [13], [14] reported triglyceride (TG) levels, five [6], [10], [11], [13], [14] reported high-density lipoprotein (HDL) levels, five [7], [10], [11], [13], [14] reported blood glucose (BG) levels, four [6], [10], [11], [14] reported low-density lipoprotein (LDL) levels, four [6], [7], [10], [13] reported systolic blood pressure (SBP), and four [7], [9], [10], [13] reported waist circumstance (WC). The definitions and methods of assessing obesity varied across the studies, with three studies [5], [6], [10] defining obesity as a BMI≥30 kg/m^2^, one study [8] using a BMI>29 kg/m^2^, one [13] using a BMI≥25 kg/m^2^ (South Korea), and one [9] using a BMI≥95th percentile (children). In all studies, natural Ad36 infection was detected by measurement of neutralizing antibodies.

Atkinson [5] reported two independent studies performed in 502 adults and 28 sets of twins, respectively, in the same paper. The mean BMI of the 502 adults (38.0 kg/m^2^) was higher than in the other studies. Trovato [6], [14] reported two studies that included patients with non-alcoholic fatty liver disease (NAFLD). The subjects of Broderick study [8] included military personnel on active duty.

### Association of Ad36 with BMI

**Figure 2:**
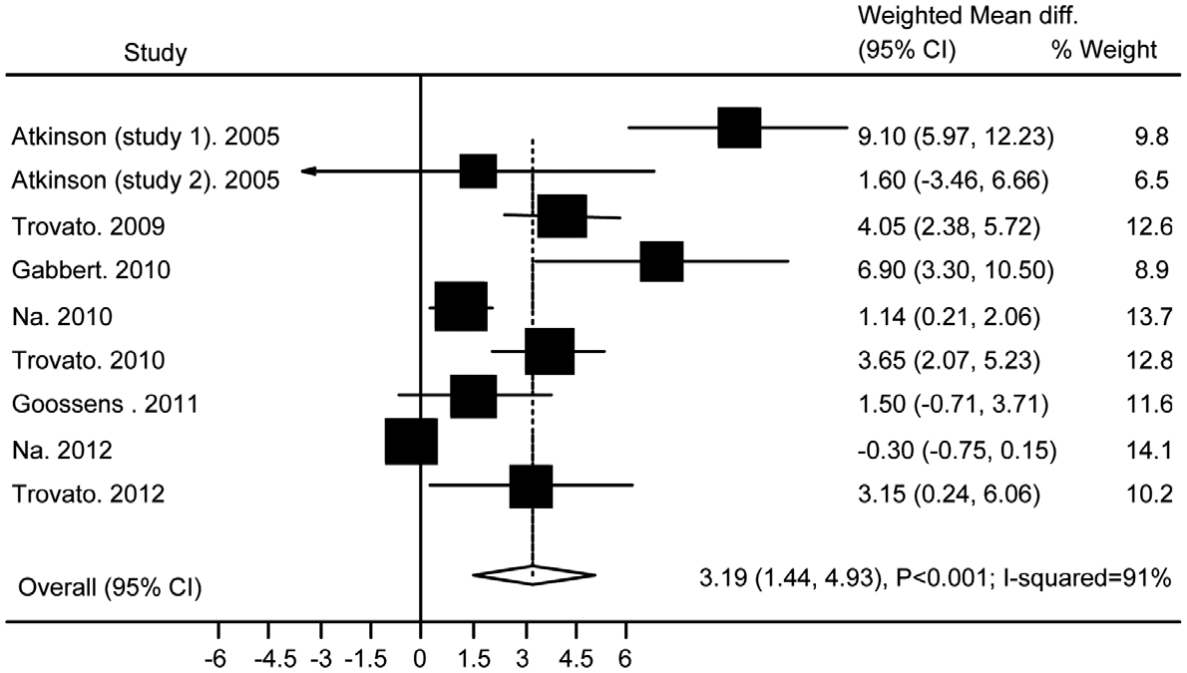
Association of Ad36 with BMI.

Of 237 potentially relevant studies, 10 cross-sectional studies with 2,870 subjects conformed to the selection criteria. None of these studies found a decrease of BMI or the risk of obesity in subjects with Ad36 infection. **Figure 2** shows the results obtained from the random effects model combining the WMDs for BMI. Across the nine studies with BMI data, the WMD for Ad36 infection compared with non-infection was 3.19 (95% CI: 1.44 to 4.93; P<0.001; I-squared = 91%). Analysis restricted to studies of adults (N = 7) showed a similar result (WMD 3.18(95% CI: 0.78 to 5.57; P = 0.009; I-squared = 92%)), but analysis of the studies done in children (N = 2) did not (P = 0.19). Analysis restricted to studies performed in the EU (N = 4) or the USA (N = 3) showed a significant difference between subjects with or without Ad36 infection (WMD 3.3 (95% CI: 2.23 to 4.34; P<0.001; I-squared = 15%) and 6.3 (95% CI: 2.41 to 10.2; P = 0.002; I-squared = 67%)), and heterogeneity was decreased. However, studies done in Asia (N = 2) did not show such a difference (P = 0.62).

Stratified analysis also showed that the pooled WMD was significant at 5.04 (95% CI: 3.18 to 6.9; P<0.001; I-squared 67%) in the mean BMI≥27 group (N = 5), whereas the pooled WMD was not significant at 0.6 (95%CI: −0.55 to 1.76; P = 0.3; I-squared 69%) in the mean BMI<27 group (N = 4).

### Association of Ad36 with Obesity

**Figure 3:**
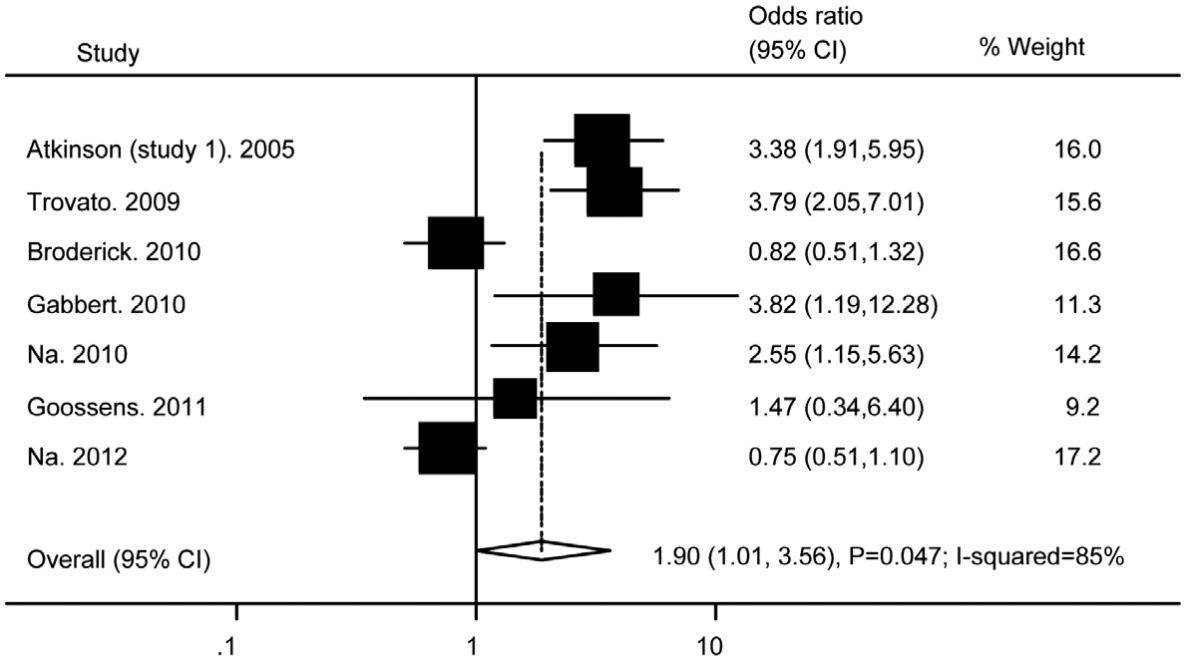
Association of Ad36 with obesity.

**Figure 3** presents the results obtained from random effects models combining the ORs for obesity. Fewer studies investigated these outcomes compared with BMI, and there was a significantly increased risk of obesity associated with Ad36 infection (OR: 1.9; (95% CI: 1.01 to 3.56; P = 0.047; I-squared = 85%). Analyses performed in the USA (N = 3), in the EU (N = 2), and in Asia (N = 2) yielded various results 2.08 (0.69 to 6.28; P = 0.2; I-squared = 88%), 3.02(1.36 to 6.68; P = 0.006; I-squared = 26%), and 1.31(0.4 to 4.37; P = 0.66; I-squared = 87%).

Sensitivity analysis was initially performed to assess the risk of obesity with the diagnostic criterion for obesity being set at BMI≥30 [4], [6], [10]. As a result, the adjusted OR of 3.3 was significant (95% CI: 2.29 to 4.79; P<0.001) and heterogeneity was reduced (I-squared = 0%).

We also performed stratified analysis by dividing the subjects into two groups with a mean BMI≥27 or a mean BMI<27. The pooled OR was significant at 3.59 (95% CI: 2.42 to 5.31; P<0.001; I-squared 0%) in the mean BMI≥27 group (N = 3) and heterogeneity was reduced, whereas the pooled OR was not significant at 1.33 (95% CI: 0.53 to 3.3; P = 0.45; I-squared 74%) in the mean BMI<27 group (N = 3).

The funnel plot, Begg’s test, and Egger’s test were used to evaluate the potential influence of publication bias on the association of Ad36 infection with obesity. The funnel plot did not show an asymmetric pattern, while Egger’s test and Begg’s test revealed no significant publication bias (all P>0.05). Moreover, univariate and multivariate meta-regression analyses revealed that the type of subject (adults or children), sex (proportion of females), and geographic region (USA, EU, or Asia) were all unrelated to the risk of obesity (all P>0.05).

### Association of Ad36 with Metabolic Markers

**Figure 4:**
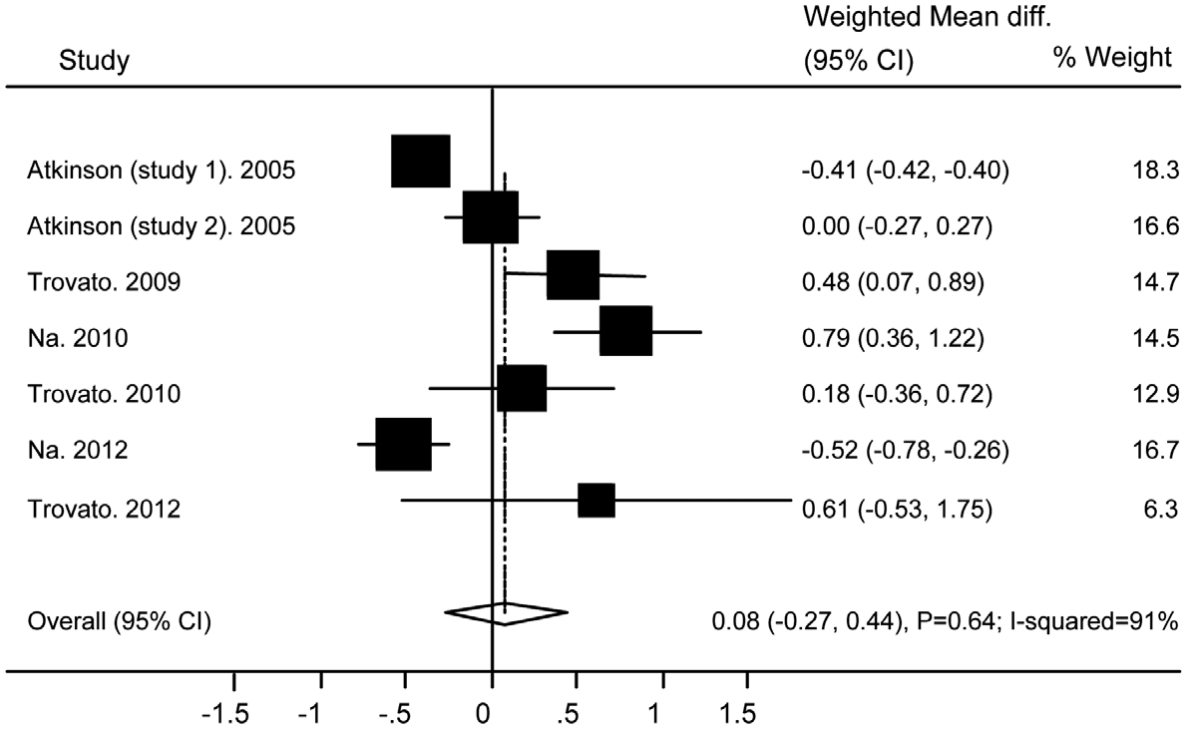
Association of Ad36 with Triglyceride.

**Figure 5:**
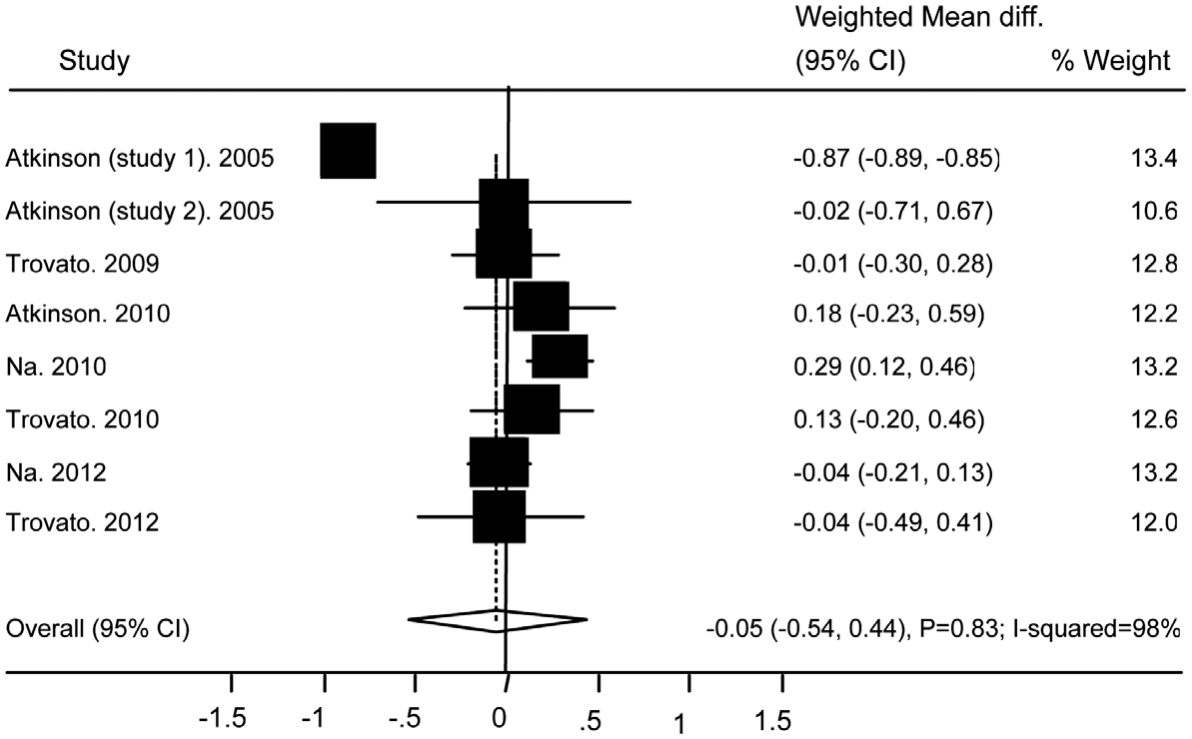
Association of Ad36 with Total Cholesterol.

**Figure 6:**
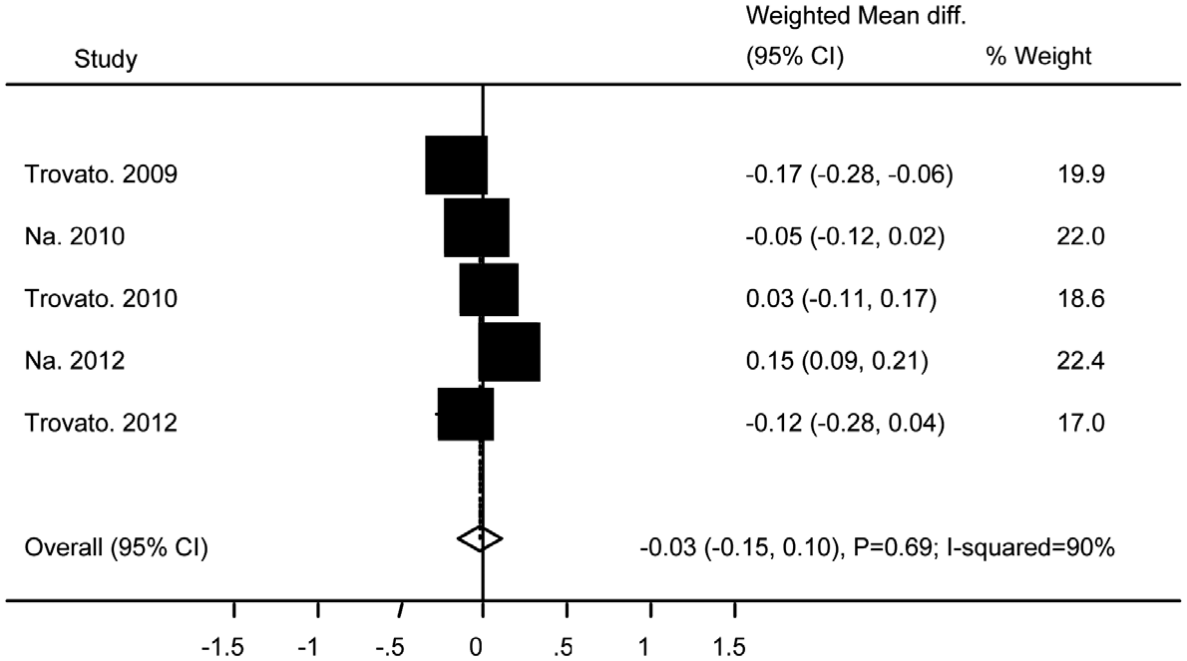
Association of Ad36 with High Density Lipoprotein.

**Figure 7:**
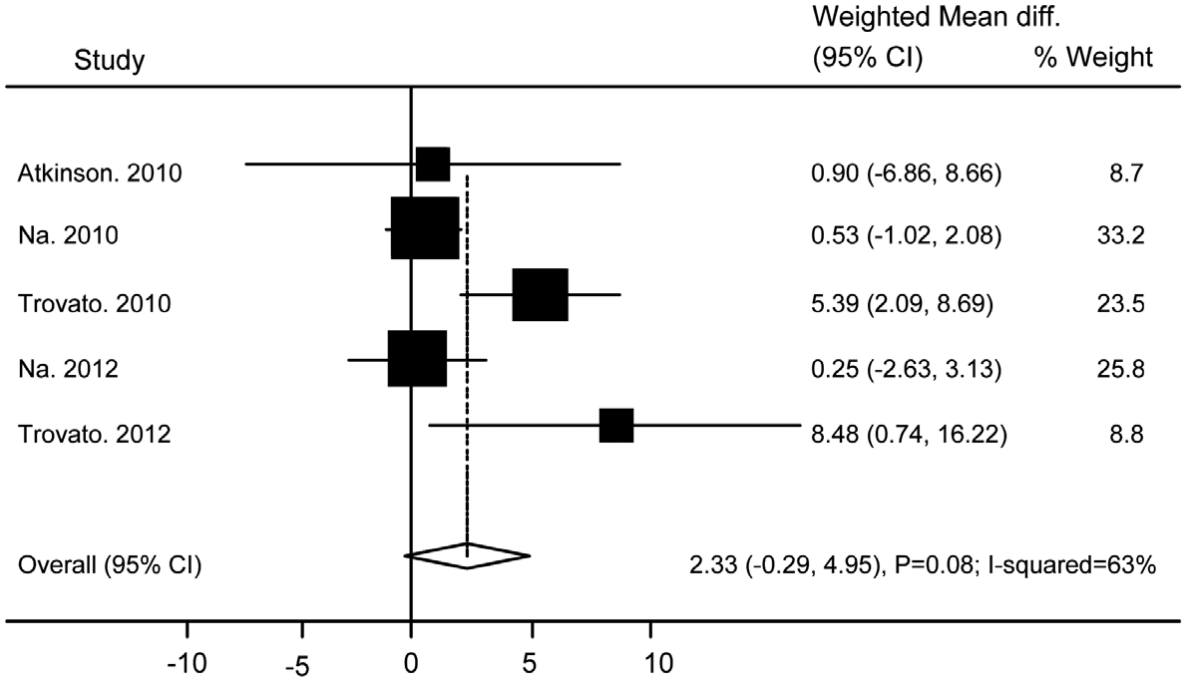
Association of Ad36 with Blood Glucose.

**Figure 8:**
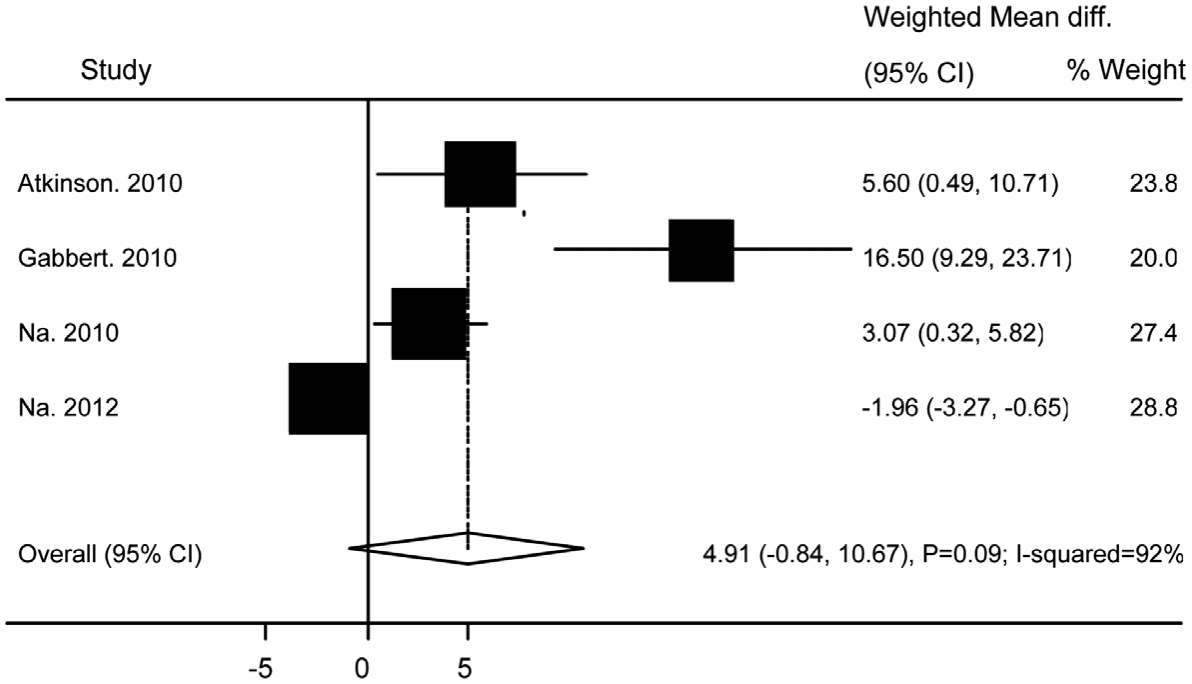
Association of Ad36 with Waist Circumstance.

**Figure 9:**
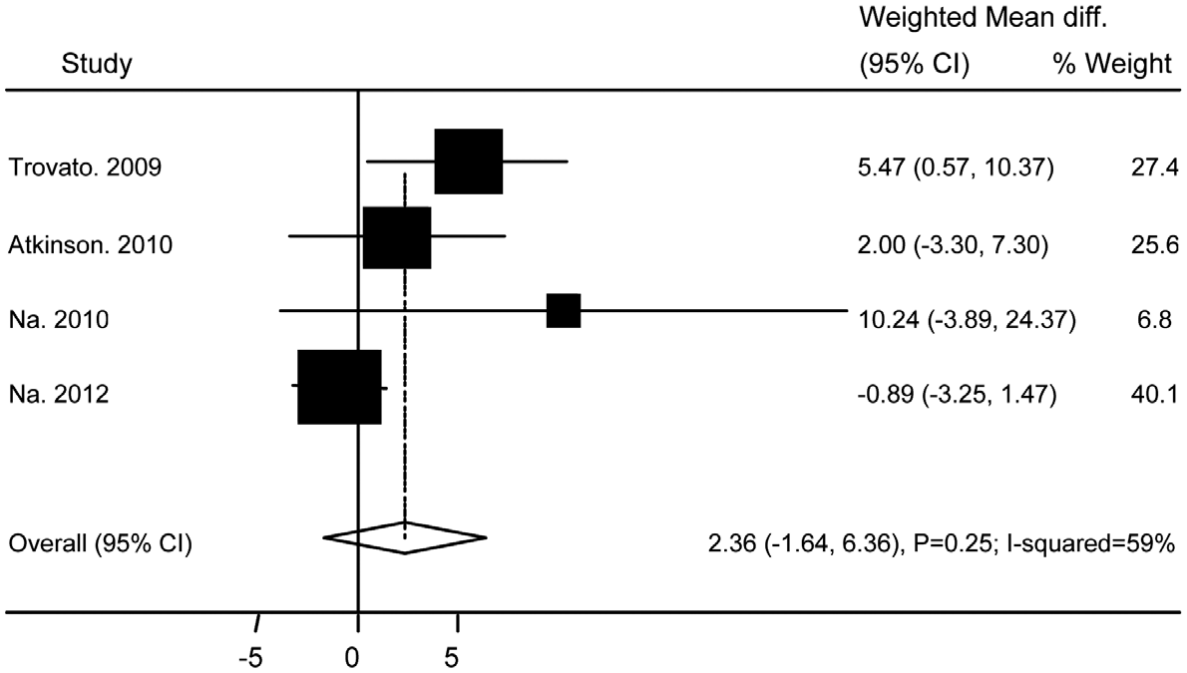
Association of Ad36 with Systolic Blood Pressure.

**Figure 10:**
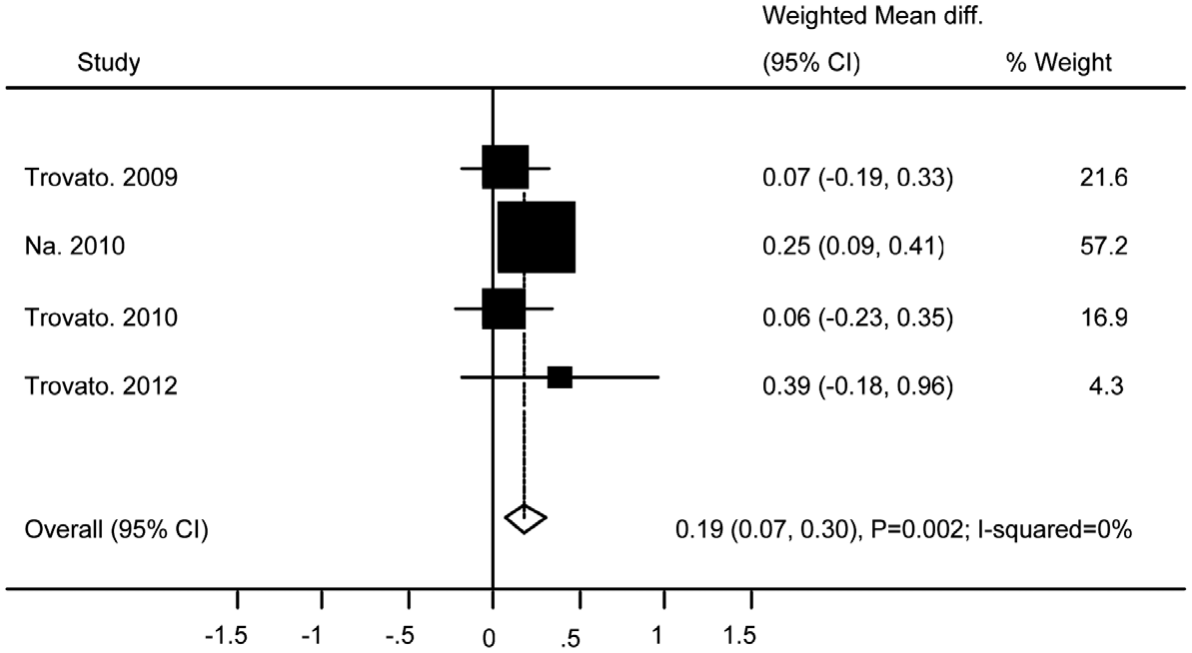
Association of Ad36 with Low Density Lipoprotein.

No significant differences were found between subjects with and without Ad36 infection in relation to TG (WMD 0.08 (95% CI: −0.27 to 0.44; P = 0.64)) (**Figure 4**), TC (WMD −0.05 (95% CI: −0.54 to 0.44; P = 0.83)) (**Figure 5**), HDL (WMD −0.03 (95% CI: −0.15 to 0.1; P = 0.69)) (**Figure 6**), BG (WMD 2.33 (95% CI: −0.29 to 4.95; P = 0.08)) (**Figure 7**), WC ((WMD 4.91 (95%CI: −0.84 to 10.7; P = 0.09)) (**Figure 8**), and SBP (WMD 2.36 (95% CI: −1.64 to 6.36; P = 0.25)) (**Figure 9**). A significant difference was found in relation to LDL, but this was subtle (WMD 0.19 (95%CI: 0.07 to 0.3; P = 0.002)) (**Figure 10**).

### Additional Analysis

Because Trovato’s studies [6], [11], [14] were all conducted in similar institutions, we carried out sensitivity analyses that each included only one of the three studies of Trovato et al. to assess the possible influence of overlapping study cohorts.

When the influence on BMI was assessed, we obtained the following results. The sensitivity analysis that included Trovato 2012 [14] and excluded Trovato 2009 [6] and Trovato 2010 [11] gave a WMD of 2.92 (95% CI: 1.0 to 4.85, P = 0.003).

In addition, the analysis that included Trovato 2010 [11] and excluded Trovato 2009 [6] and Trovato 2012 [14] gave a WMD of 3.04 (95% CI: 1.09 to 4.99, P = 0.002).

Furthermore, the sensitivity analysis including Trovato 2009 [6] and excluding Trovato 2010 [11] and Trovato 2012 [14] yielded a WMD of 3.12 (95% CI 1.12 to 5.11, P = 0.002).

All three analyses showed a significantly higher BMI in subjects with Ad36 infection than in those without infection. The results were also similar to that of our overall analysis (WMD: 3.19), which was done before conducting the sensitivity analyses.

No effect of obesity was detected by the primary analysis because obesity was only described in one report by Trovato [6].

Subsequently, we conducted sensitivity analyses of other metabolic markers reported in two or more of Trovato’s papers, including TG, TC, HDL, BG, and LDL.

The results of these analyses showed that there were no significant differences between subjects with or without Ad 36 infection in relation to four metabolic markers (TG, TC, HDL, and BG) (all P>0.05), corresponding to the outcome of our primary analysis.

In the case of LDL, the sensitivity analysis that included Trovato 2012 [14] and excluded Trovato 2009 [6] and Trovato 2010 [11] gave a WMD of 0.26 (95% CI: 0.11 to 0.41, P = 0.001), while the analysis including Trovato 2010 [11] and excluding Trovato 2009 [6] and Trovato 2012 [14] gave a WMD of 0.2 (95% CI: 0.03 to 0.36, P = 0.02).

Moreover, the analysis that included Trovato 2009 [6] and excluding Trovato 2010 [11] and Trovato 2012 [14] yielded a WMD of 0.19 (95% CI: 0.02 to 0.36, P = 0.03).

These results for LDL were not significantly different to the result of our primary analysis performed prior to the sensitivity analyses (WMD: 0.19 [95% CI: 0.07 to 0.3]), although a very slight increase of WMD to 0.26 was observed.

## Discussion

This meta-analysis of 10 studies from around the world demonstrated that Ad36 infection was associated with the risk of obesity and weight gain (an estimated BMI increase of 3.19 kg/m^2^), but was not associated with abnormal metabolic markers including waist circumstance. It suggests that Ad36 infection is more associated with accumulation of subcutaneous fat than that of visceral fat. Sensitivity analysis showed that our results were relatively robust, while meta-regression analysis indicated that the findings were not affected by differences of geographical region or age. When stratified analysis was done by dividing the subjects into two groups with a mean BMI≥27 or BMI<27, we found that infection with Adenovirus 36 was more closely related to obesity and weight gain in the heavier subjects compared with the lighter subjects. These results have certain implications for the management of obesity, especially in view of the recent exponential increase in its prevalence.

Most of the studies included in our meta-analysis were from a few groups of authors, so it was important to determine whether or not the subjects were from overlapping cohorts. Therefore, we investigated the studies of Atkinson et al., Na et al., and Trovato et al., which had to potential to include overlapping populations.

Atkinson et al. authored two papers about the relation between Ad36 and obesity (Atkinson 2005 [5] and Atkinson 2010 [7]). Atkinson 2005 [5] reported two observational studies in adults (study 1 involved 360 obese persons and 142 non-obese persons, while study 2 enrolled 28 sets of twins). These two studies were described by Atkinson as individually independent cohort studies conducted in different regions. In addition, we asked Atkinson about the study cohorts via e-mail, and confirmed that these studies were not conducted in the same cohort. On the other hand, Atkinson 2010 [7] reported a study conducted in children. Therefore, we judged that the three study populations reported in these two papers were all different cohorts.

Na et al. also authored two papers (Na 2010 [10] and Na 2012 [13]). Na 2010 [10] reported a study conducted in children, while Na 2012 [13] reported a study performed in adults. Accordingly, these studies were considered to be unlikely to involve the same cohort. In fact, we confirmed that these two studies were not conducted in the same cohort after contacting Na by e-mail.

Trovato et al. authored three papers about Ad36 and obesity (Trovato 2009 [6], Trovato 2010 [11], and Trovato 2012 [14]). These papers did not state that the subjects in each study were from the same cohort, but the studies were conducted at similar institutions. Also, both Trovato 2010 [11] and Trovato 2012 [14] described studies conducted in patients with NAFLD, so these two studies could have been conducted in the same cohort.

However, we performed sensitivity analyses that each included only one of the three studies of Trovato et al. and obtained similar results to those of the primary analysis for all metabolic markers, including obesity and BMI.

Although this meta-analysis included several reports by the same groups, most of the actual studies were conducted in independent cohorts. Also, the results of sensitivity analyses performed on Trovato’s studies that could have included the same cohort showed that there was no influence on the outcome of our primary analysis. In other words, the results were consistent with our conclusion that Ad36 infection can significantly influence obesity and BMI, while it has little effect on other metabolic markers.

### Mechanism of the Relation between Ad36 and Obesity

Recent studies have shown that Ad36 infection increases adiposity in several animal models including marmosets, rats, mice, and chickens [19]–[22]. Ad36 infection significantly reduces serum cholesterol and triglyceride concentrations compared with those in uninfected controls. Interestingly, longitudinal studies performed in monkeys [21] have revealed an increase of body weight by about 15% and a 29% reduction of serum cholesterol after natural Ad36 infection.

Several mechanisms have been postulated to explain the association between Ad36 infection and obesity. Results of both in vivo and in vitro investigations have revealed that Ad36 infection accelerates the differentiation of preadipocytes into adipocytes and their proliferation in studies of 3T3-L1 cells and human preadipocytes [23]–[25]. Ad36 infection also raises the lipid content of fat cells by promoting the uptake of lipids and glucose, which increases cellular lipid levels by stimulation of de novo lipogenesis [25], [26].

### Limitations

Our study had several limitations. First, we only reviewed English-language reports, which could cause selection bias. Second, substantial heterogeneity was observed among the studies even after sensitivity analysis, suggesting that the different epidemiological characteristics (e.g., prevalence of Ad36 infection or different diagnostic criteria for obesity) of the patient populations contributed to this heterogeneity to some extent. This heterogeneity means that there was a wide range of plausible risk estimates, but we found no evidence to suggest that Ad36 infection is associated with a lower risk of obesity or a lower BMI. Although differences of the geographical region and age did not explain the heterogeneity in meta-regression analysis, it must be remembered that it is impossible to avoid the influence of measured (and unmeasured) confounders in observational studies.

Moreover, this meta-analysis only included case-control studies, so a causal relation with obesity could not be proven. Ideally, these results should be confirmed in prospective clinical trials, but ethical considerations preclude experimental infection of humans with candidate viruses to unequivocally define their contribution to obesity. Accordingly, we cannot rule out the possibility that the association of Ad36 with obesity results from greater susceptibility of obese individuals to infection compared with non-obese individuals [27]. However, a causal relationship between adenovirus 36 infection and obesity has been demonstrated in animals [19]–[22].

Third, there was limited information about the use of medications that may have contributed to obesity, such as insulin, sulfonylureas, and antipsychotics [28], [29]. Cessation of smoking is also associated with weight gain [30]. Although uncommon, some obese patients have endocrine disorders such as Cushing’s syndrome [31].

Even with such limitations, these observational studies provided useful evidence regarding the potential influence of Ad36 infection on obesity. Patients and physicians need to consider the possible increased risk of weight gain associated with Ad 36 infection.

### Conclusions

Ad36 infection was associated with the risk of obesity and weight gain, but was not associated with abnormal metabolic markers. The relationship between Ad36 infection and obesity should be assessed further by well-designed prospective studies to gain a better understanding of whether this virus plays a role in the etiology of human obesity.

## Supporting Information

Checklist S1 **PRISMA Checklist.**
(DOC)
